# Stimuli-responsive HBPS-*g*-PDMAEMA and its application as nanocarrier in loading hydrophobic molecules

**DOI:** 10.3762/bjoc.12.92

**Published:** 2016-05-10

**Authors:** Yongsheng Chen, Li Wang, Haojie Yu, Zain-Ul-Abdin, Ruoli Sun, Guanghui Jing, Rongbai Tong, Zheng Deng

**Affiliations:** 1 State Key Laboratory of Chemical Engineering College of Chemical and Biological Engineering, Zhejiang University, Hangzhou, 310027, China

**Keywords:** amphiphilic polymer, hyperbranched polystyrene, phase transition, poly[2-(dimethylamino)ethyl methacrylate] (PDMAEMA), stimuli-responsive nanocarriers

## Abstract

The topic of stimuli-responsive nanocarriers for loading guest molecules is dynamic. It has been widely studied in applications including drug controlled release, smart sensing, catalysis, and modeling. In this paper, a graft copolymer (hyperbranched polystyrene)-*g*-poly[2-(dimethylamino)ethyl methacrylate] (HBPS-*g*-PDMAEMA) was synthesized and characterized by ^1^H NMR and GPC. It was observed that the star-like HBPS-*g*-PDMAEMA formed aggregates in aqueous solution. The influence of polymer concentration, ionic strength and pH value on the aggregates in aqueous solution was investigated by using UV–vis spectroscopy and DLS analysis. The results showed that size of aggregates was affected by a corresponding stimulus. In addition, the loading ability of HBPS-*g*-PDMAEMA aggregates was investigated by using pyrene or Nile red as the model guest molecules by using UV–vis and fluorescence spectroscopy. The results showed that HBPS-*g*-PDMAEMA aggregates were capable to encapsulate small hydrophobic molecules. These newly prepared HBPS-*g*-PDMAEMA nanocarriers might be used in, e.g., medicine or catalysis.

## Introduction

Stimuli-responsive polymers have attracted much attention due to their broad applications including drug controlled release [1–5], sensing [6–7] and 4D printing [8–9]. Nanocarriers prepared from amphiphilic stimuli-responsive polymers are promising candidates for transporting hydrophobic guest molecules such as anticancer or antitumor drugs. In the cases of polymeric nanocarriers with responsive shell and stable core, the external hydrophilic shell makes these nanocarriers soluble in water and affords stimuli-responsive properties. The interior hydrophobic core acts as a container for guest molecules and affords protection [10–12]. It is urgent to study their stimuli-responsive properties and to develop suitable nanocarriers for certain applications.

Poly[2-(dimethylamino)ethyl methacrylate] (PDMAEMA) is a well-known stimuli-responsive polymer that responds to changes in temperature, pH and ionic strength [13–14]. Among temperature-responsive polymers, PDMAEMA is an important material, as its low critical solution temperature (LCST) is close to the human body temperature. The stimuli-responsive polymers can be prepared by grafting or blocking hydrophilic PDMAEMA chains on a hydrophobic chain. These amphiphilic polymers may aggregate in water to form nanoparticles having stimuli-responsive properties. Different hydrophobic segments with different microstructures give the aggregates adjustable properties that can be used for various applications under different conditions. The study of the encapsulation of hydrophobic molecules into amphiphilic nanocarriers is important for cancer or tumor therapies because many of the newly synthesized efficient and useful drugs are insoluble in water.

Hyperbranched or star-like polymers have become attractive to academia and industry in recent years due to their special chemical and physical properties. In comparison with their linear counterpart, these polymers afford promising applications such as drug delivery [15–17], gene delivery [18–21] and catalysis [22–23]. Polystyrene is a biocompatible material with low polarity. It has been found that a hydrophobic polymer segment with low polarity was helpful in drug delivery systems and in the protection of drugs from degradation [12,24]. Star-like amphiphilic polymers can be prepared by grafting hydrophilic stimuli-responsive chains on hyperbranched polystyrene. Chen reported a star-like polymer HBPS-*g*-PNIPAM in which HBPS was prepared by click chemistry to load small hydrophobic molecules [25]. However, its preparation needs specially designed monomers, the preparation of which is tedious and time-comsuming. Hyperbranched polystyrene (HBPS) prepared through atom transfer radical self-condensing vinyl polymerization (AT-SCVP) was firstly reported in 1996 [26]. It used vinylbenzyl chloride (VBC), which is an important commercially available monomer and its industrial synthesis was started from 1957, as monomer with a self-initiation site [27]. Produced HBPS is tethered with lot of peripheral chlorine atoms available for further modification or functional chain extension.

Here, we prepared a hyperbranched graft copolymer HBPS-*g*-PDMAEMA. HBPS segments behaved as hyperbranched topology support and also as hydrophobic segment to give HBPS-*g*-PDMAEMA aggregates stability in aqueous solution. The PDMAEMA segments behaved as functional chains to afford stimuli-responsive properties. The phase-transition behavior of the polymer aggregates in aqueous solution was studied. The size of the polymer aggregates under different conditions (polymer concentration, ionic strength and pH value) was investigated. The loading ability for small hydrophobic molecules was studied. To the best of our knowledge, this is the first report to prepare HBPS-containing stimuli-responsive materials for loading small molecules.

## Results and Discussion

### Synthesis and characterization of HBPS and HBPE-*g*-PDMAEMA

**Figure 1:**
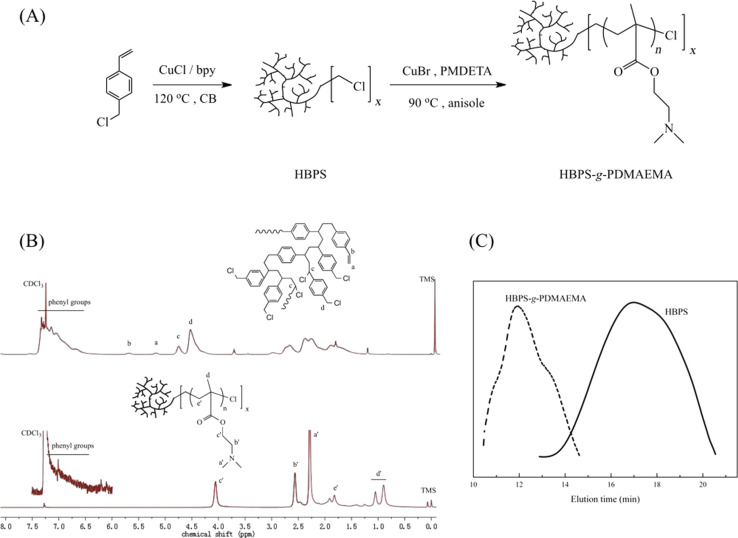
(A) The synthetic routes to HBPS and HBPS-*g*-PDMAEMA; (B) ^1^H NMR spectra of HBPS and HBPS-*g*-PDMAEMA and (C) GPC curves of HBPS and HBPS-*g*-PDMAEMA.

The target polymer HBPS-*g*-PDMAEMA was prepared through AT-SCVP and following ATRP strategies as shown in Figure 1A. HBPS with hyperbranched topology was prepared through atom transfer radical self-condensing vinyl polymerization (AT-SCVP) by using vinylbenzyl chloride (VBC), a commercially available monomer, as monomer and self-initiator. The feeding ratio of VBC/CuCl/2,2-bipyrene (bpy) was 10:1:2. The ^1^H NMR spectrum of HBPS showed peaks around 5.7 and 5.2 ppm attributed to double bonds [26–27] as shown in Figure 1B. For AT-SCVP, the polymerization was initiated by benzyl chloride of VBC. It was found that each polymer chain was tethered with a double bond and chloride groups. Each monomer inserted into the polymer chain has one initiate site to start propagation of the side chains, which leads to the formation of hyperbranched topology. The broad peak from 4.1 to 4.9 ppm was attributed to the methylene and methine protons adjacent to chlorine atoms, in which the split peak around 4.6 ppm was attributed to methylene and the split peak around 4.8 ppm was attributed to methine. The broad peak from 6.0 ppm to 7.5 ppm was attributed to phenyl groups. The obtained HBPS was used as macro initiator to polymerize DMAEMA through ATRP. The comparison of GPC curves in Figure 1C showed that the curve of the graft copolymer was shifted to less elution time. The GPC results show *M_n_* of HBPS to be 4.4 kg/mol with a molecular weight distribution of 1.85 and *M_n_* of HBPS-*g*-PDMAEMA is 151.3 kg/mol with a molecular weight distribution of 2.84. The molecular weight of HBPS-*g*-PDMAEMA is bigger than HBPS, which demonstrated that HBPS-containing chlorine atoms successfully initiated the ATRP of DMAEMA, which resulted in HBPS-*g*-PDMAEMA. Figure 1B shows the ^1^H NMR spectrum of HBPS-*g*-PDMAEMA. The structure of PDMAEMA was clearly confirmed according to literature [28] and the signal of HBPS segment was weak and appeared between 6.0 and 7.5 ppm [29]. The results of ^1^H NMR and GPC measurements showed that the target polymer HBPS-*g*-PDMAEMA was successfully prepared.

### The influence of temperature on HBPE-*g*-PDMAEMA aggregates

**Figure 2:**
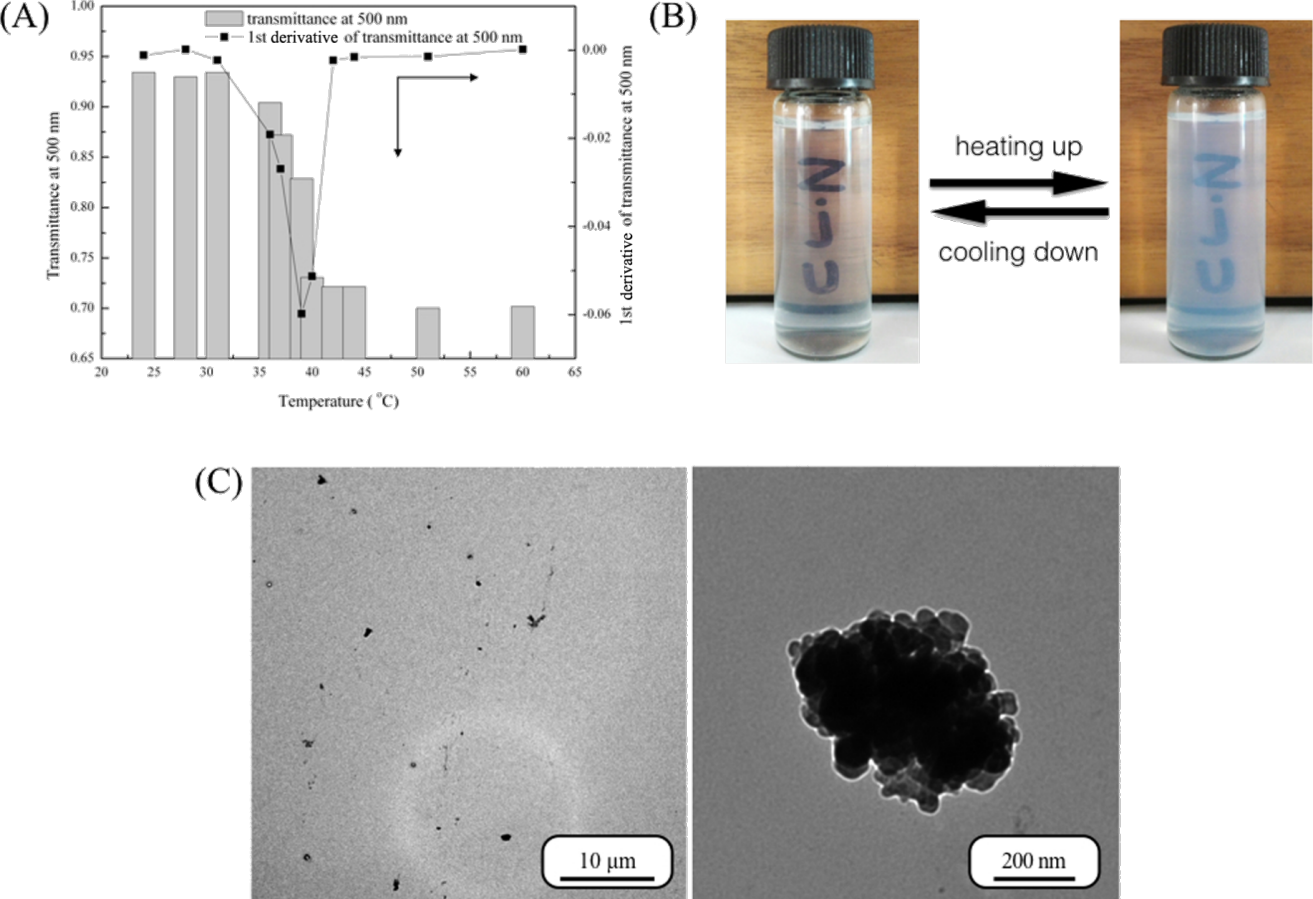
(A) The transmittance of HBPS-*g*-PDMAEMA aqueous solution of 10 mg/mL (pH 7); (B) pictures of aqueous solution of HBPS-*g*-PDMAEMA at 10 mg/mL below and above the LCST and (C) TEM images of HBPS-*g*-PDMAEMA aggregates.

The PDMAEMA polycation becomes soluble in water due to the protonation of amine groups and hydrogen bonding with water molecules. Furthermore, PDMAEMA is sensitive to temperature, pH and ionic strength. The influence of temperature on PDMAEMA is important, because the LCST of PDMAEMA in water is near the human body temperature; a fact that might be exploited in drug delivery systems. We checked the light transmittance of the HBPS-*g*-PDMAEMA aqueous solution at a wavelength of 500 nm and a concentration of 10 mg/mL by using a UV–vis spectrophotometer equipped with a water bath, as shown in Figure 2A.

In this case, pH value and ionic strength were not adjusted and deionized water was directly used to prepare the samples. The transmittance was constant around 0.95 from 25 to 30 °C. As the temperature was increased, the transmittance was decreased and reached a stable value at 40 °C. The curve of the first derivative showed that the transmittance decreased dramatically at a temperature of about 39 °C, which is the LCST of HBPS-*g*-PDMAEMA in water at a concentration of 10 mg/mL. Figure 2B shows the phase transition of HBPS-*g*-PDMAEMA in water. When the temperature is lower than the LCST, the solution is clear, which demonstrates that the PDMAEMA segment is soluble in water. When the temperature is higher than the LCST, the solution becomes opaque, which demonstrates that the PDMAEMA chains become partially insoluble. In aqueous solution, the phase inversion depends on the formation or breakdown of hydrogen bonds between polymer chains and water molecules as well as entropically driven effects. Figure 2C shows TEM images of HBPS-*g*-PDMAEMA aggregates. It was observed that the morphology of aggregates is inhomogeneous and the aggregates were composed of clusters of smaller units. These aggregates were soluble in water due to water soluble PDMAEMA chains. The aggregates were also stable enough because the incorporated HBPS segment is an excellent hydrophobic polymeric chain.

**Figure 3:**
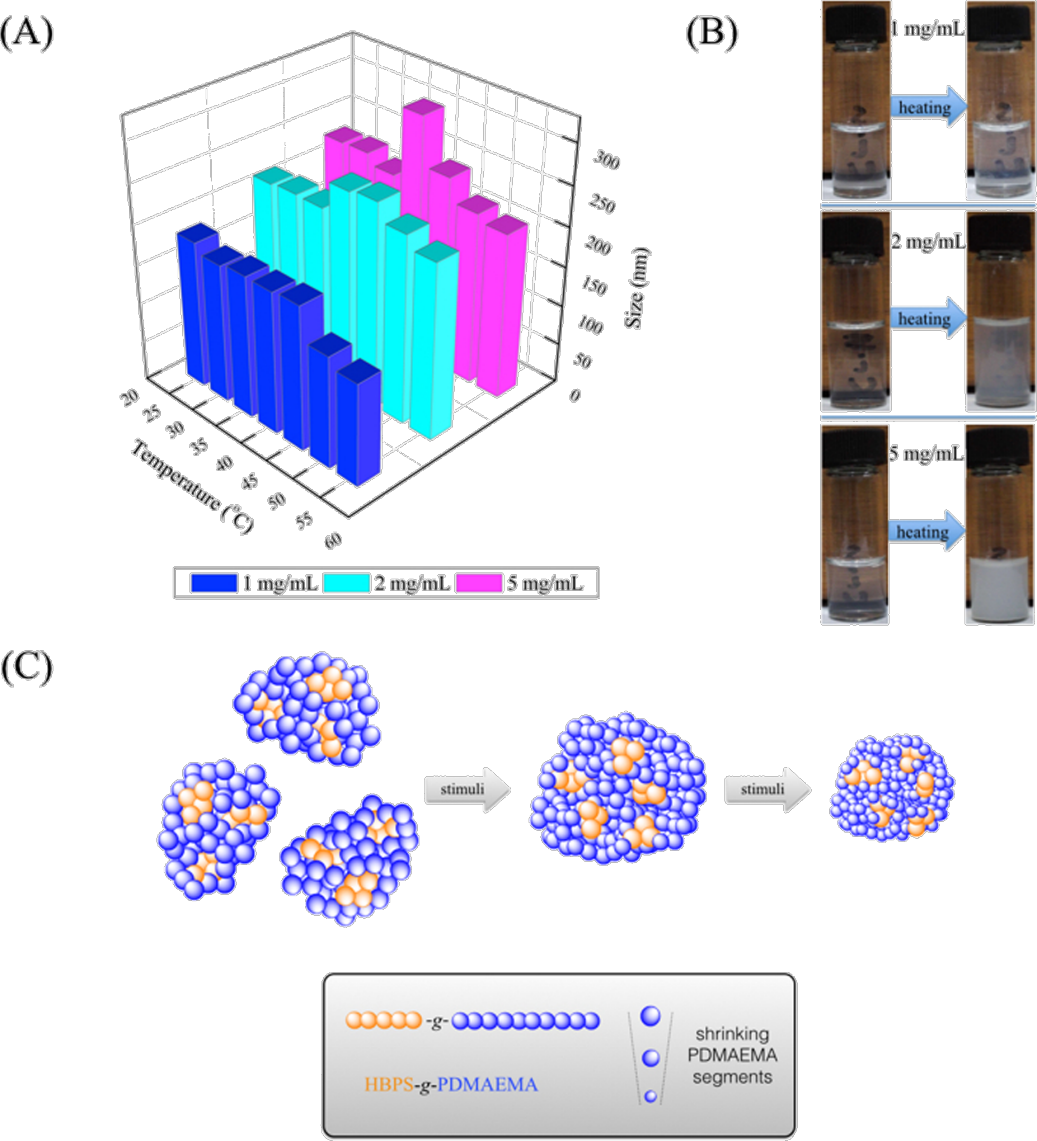
(A) The effect of HBPS-*g*-PDMAEMA concentration on the size of aggregates (pH 7); (B) photographs of phase inversion of HBPS-*g*-PDMAEMA at different concentrations of 1, 2 and 5 mg/mL in aqueous solutions and (C) the illustrated mechanism of changed sizes of HBPS-*g*-PDMAEMA aggregates in aqueous solution at different concentrations.

To further investigate the behavior of HBPS-*g*-PDMAEMA aggregates in water, DLS measurements were performed and the change in aggregate size as a function of temperature and concentration were recorded. The solutions were prepared by dissolving HBPS-*g*-PDMAEMA in water. The results of the DLS measurements are shown in Figure 3A. Higher polymer concentrations led to larger aggregate sizes at each temperature. The HBPS-*g*-PDMAEMA aggregates at higher concentrations, such as 2 and 5 mg/mL, showed the maximum size at temperatures of 35–40 °C, close to the LCST of 10 mg/mL HBPS-*g*-PDMAEMA aggregates in water at 39 °C. Above 40 °C, the size of HBPS-*g*-PDMAEMA aggregates decreased with increasing temperature. The solution with concentration 1 mg/mL showed no obvious increase close to the LCST, but at higher temperatures the aggregate sizes become smaller. Figure 3B shows that these three samples all exhibit phase-inversion behavior. When the temperature was higher than the LCST, the solution became opaque. Figure 3C showed the proposed mechanism to explain the size change with temperature. At a temperature below LCST, the aggregates were formed from self-assembly of amphiphilic graft copolymer HBPS-*g*-PDMAEMA. When the temperature reached the LCST, the PDMAEMA chains became insoluble in water and the relatively higher concentration made the PDMAEMA chains of two aggregates coil with each other, which led to bigger aggregates. As the temperature was increased further, PDMAEMA chains started to shrink and the size of the aggregates decreased.

### The effect of ionic strength on the aggregation behavior of HBPS-*g*-PDMAEMA

**Figure 4:**
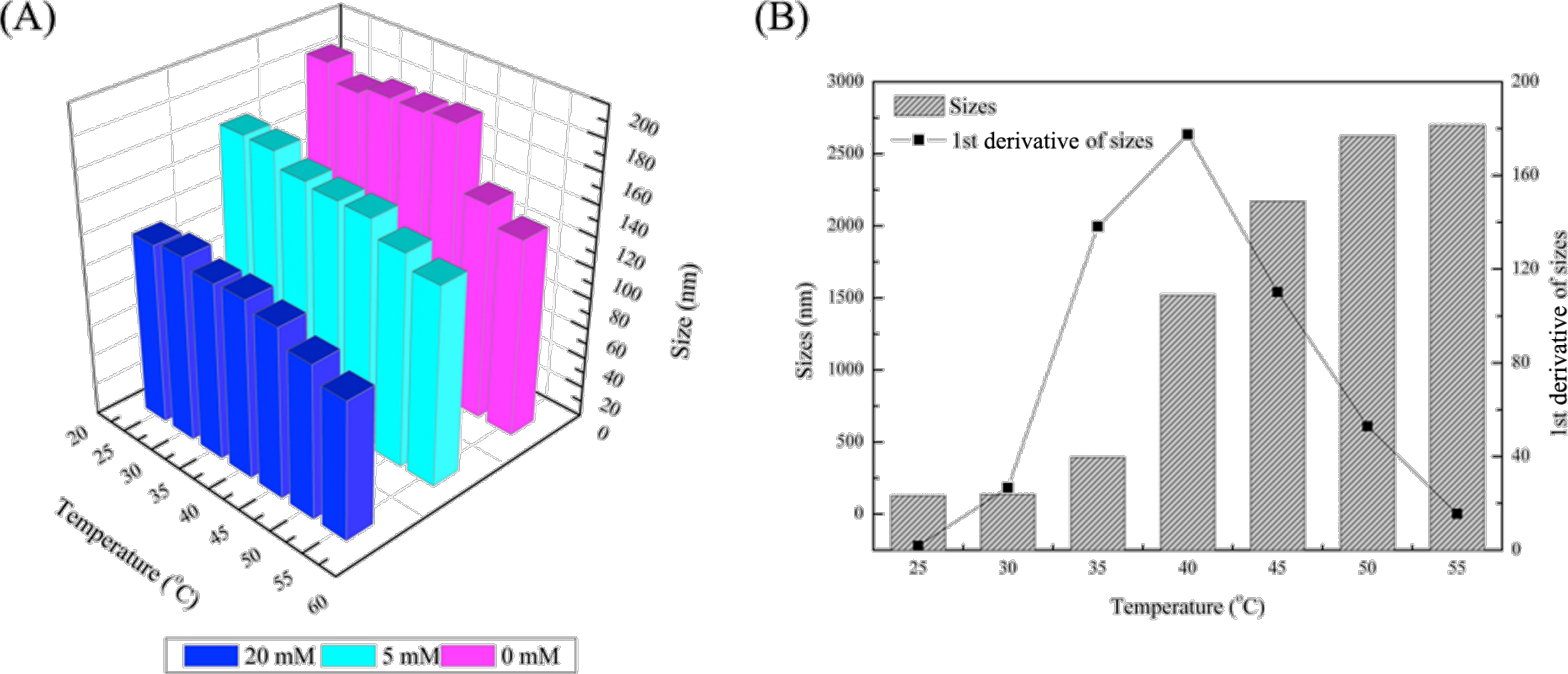
The sizes of 1 mg/mL HBPE-*g*-PDMAEMA aggregates in aqueous solutions with different concentration of NaCl (A) 0, 5 and 20 mM; and (B) 154 mM (pH 7).

The electrostatic interaction is a major factor to influence the behavior of PDMAEMA in aqueous solution. So in this work, the influence of ionic strength, which affects the electrostatic interactions, was studied. In this case, pH was not adjusted and deionized water was used to prepare buffer solutions of different ionic strengths. Figure 4A shows the size of 1 mg/mL HBPS-*g*-PDMAEMA aggregates at NaCl concentrations of 0, 5 and 20 mM as a function of the temperature. The size of polymer aggregates decreased with increasing temperature either in deionized water or aqueous solution with relatively low concentrations of NaCl, and there was no maximum point. This is because higher concentrations of NaCl lowered the repulsive electrostatic interactions between PDMAEMA chains. So, higher concentrations of NaCl led to smaller aggregation sizes. The change of size was caused from the tendency to insolubility of PDMAEMA segments at higher temperature, but for the sample with 154 mM NaCl, the trend was very different. The size increased with heating and reached above 500 nm at 40 °C to even 2500 nm at 55 °C. The concentration of NaCl was sufficiently high to break the electrostatic interaction of PDMAEMA segments, so aggregation occurred and large sized aggregates were formed.

### Effect of the pH value on the phase-transition of HBPS-*g*-PDMAEMA

**Figure 5:**
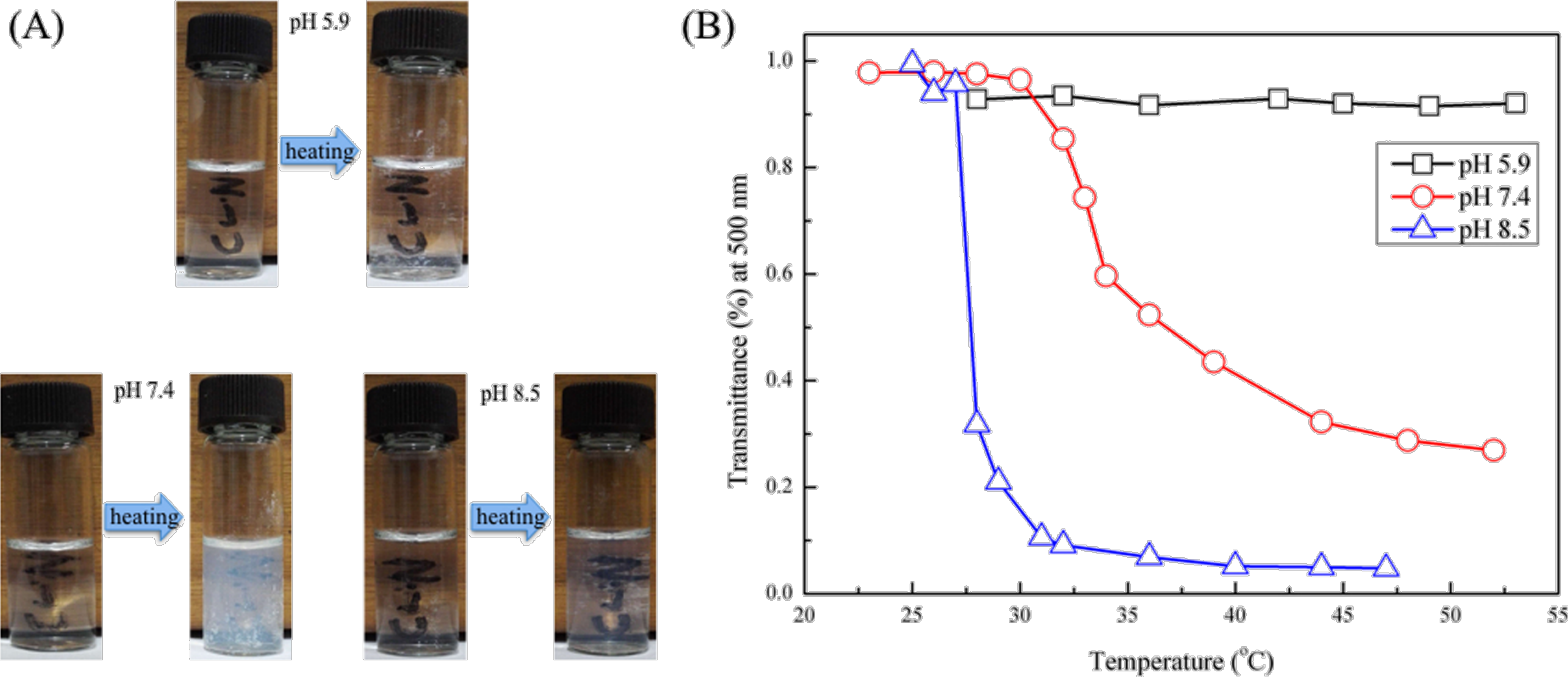
(A) Photographs of phase inversion of 1 mg/mL HBPS-*g*-PDMAEMA in aqueous solution with different pH values and (B) the transmittance at 500 nm of HBPS-*g*-PDMAEMA in aqueous solution with different pH values.

The phase-inversion behavior of HBPS-*g*-PDMAEMA depends on the hydrophobicity of PDMAEMA chains. The protonation of the tethered amine groups and the hydrogen bonding with water molecules are the major factors. In this work, the effect of pH on the phase-inversion behavior of HBPS-*g*-PDMAEMA is also investigated. In this case, the change in ionic strength was ignored and it was not adjusted by using NaCl due to the small amounts of added NaOH or HCl. The solution samples were prepared by the addition of certain amounts of polymer in aqueous solutions with certain pH values of 5.9, 7.4 and 8.5. The concentrations of all the aqueous HBPS-*g*-PDMAEMA solutions were 1 mg/mL. Figure 5A shows photographs of each sample solution before and after heating. The solution with pH 5.9 showed no obvious change before and after heating. The solutions with pH 7.4 and 8.5 showed phase-inversion and become turbid after heating. To further demonstrate phase-inversion behavior, transmittance of the solution was recorded at 500 nm and at different temperatures by using UV–vis measurements as shown in Figure 5B. The transmittance of the solution with pH 5.9 showed no turning-point, but the transmittance of the solutions with pH 7.4 and 8.5 showed obvious turning points with increasing in temperatures. The acidic environment could protonate the amine groups and make the PDMAEMA molecules soluble in water even at temperature above its LCST. Furthermore, the LCST of the solution with pH 8.5 was lower than that of the solution with pH 7.4. This may be caused by a high degree of amine deprotonation. The high pH value led to the insolubility of PDMAEMA chains, which resulted in the phase inversion of HBPS-*g*-PDMAEMA aggregates.

**Figure 6:**
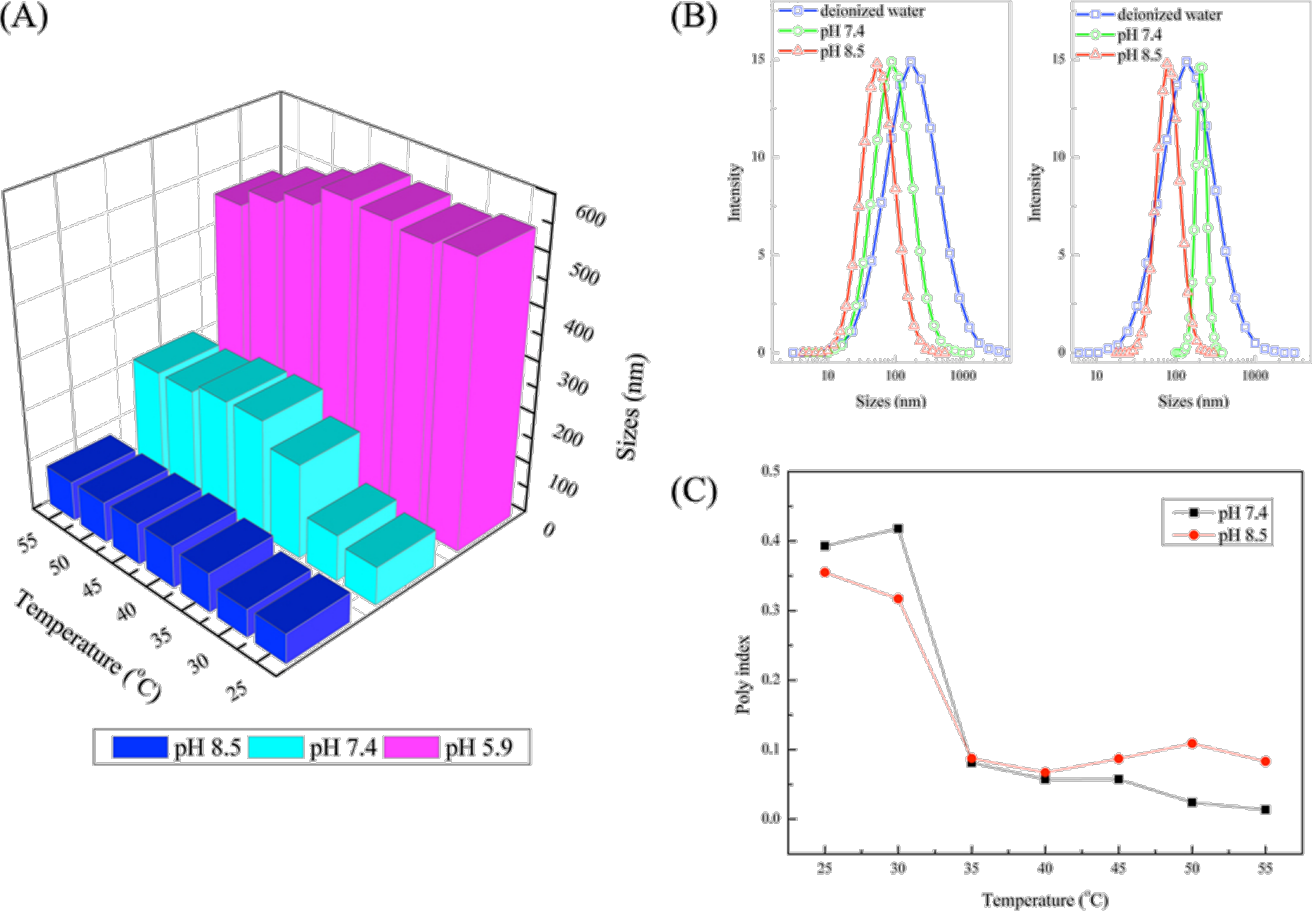
The effect of pH on: (A) the size of 1 mg/mL HBPS-*g*-PDMAEMA aggregates; (B) the DLS results of 1 mg/mL HBPS-*g*-PDMAEMA aggregates in aqueous solution with pH 7.0, 7.4 and 8.5 at 30 and 50 °C; and (C) PDI of HBPS-*g*-PDMAEMA as function of the temperature in aqueous solutions with pH 7.4 and 8.5.

The effect of pH on the size of HBPS-*g*-PDMAEMA aggregates was investigated by DLS analysis, the results of which are shown in Figure 6A. At higher pH, a lower degree of protonation occurred, which led to a smaller size of HBPS-*g*-PDMAEMA aggregates in aqueous solution. Figure 6B shows DLS results of HBPS-*g*-PDMAEMA aggregates in aqueous solution at pH 7.0, 7.4 and 8.5 at 30 and 50 °C. In deionized water, a broad size distribution of HBPS-*g*-PDMAEMA aggregates was obtained. The size of many aggregates in deionized water was higher than 500 nm. It is very interesting that in basic environment, the aggregates showed a lower distribution than in deionized water. The effect of temperature and pH on polydispersity index (PDI) of HBPS-*g*-PDMAEMA aggregates was studied, as shown in Figure 6C. As temperature was increased from 35 °C, the PDI of these two samples sharply decreased below 0.1, which means that the aggregates under these conditions may be uniform. The PDI and the size of nanoparticles used for biomedicine are important. For the human body, the size of these particles should be about 50–200 nm which could be excreted without any toxic effects. Larger nanoparticles may eliminate quickly from human body and smaller nanoparticles may block blood capillaries. In this work, pH 7.4 was similar to the pH of human blood and pH 8.5 was in the range of some secretion. In these pH, HBPS-*g*-PDMAEMA aggregates showed a narrow distribution which means that these stimuli-responsive polymers may be suitable for medicinal use.

### The encapsulation of hydrophobic molecules in HBPS-*g*-PDMAEMA aggregates

**Figure 7:**
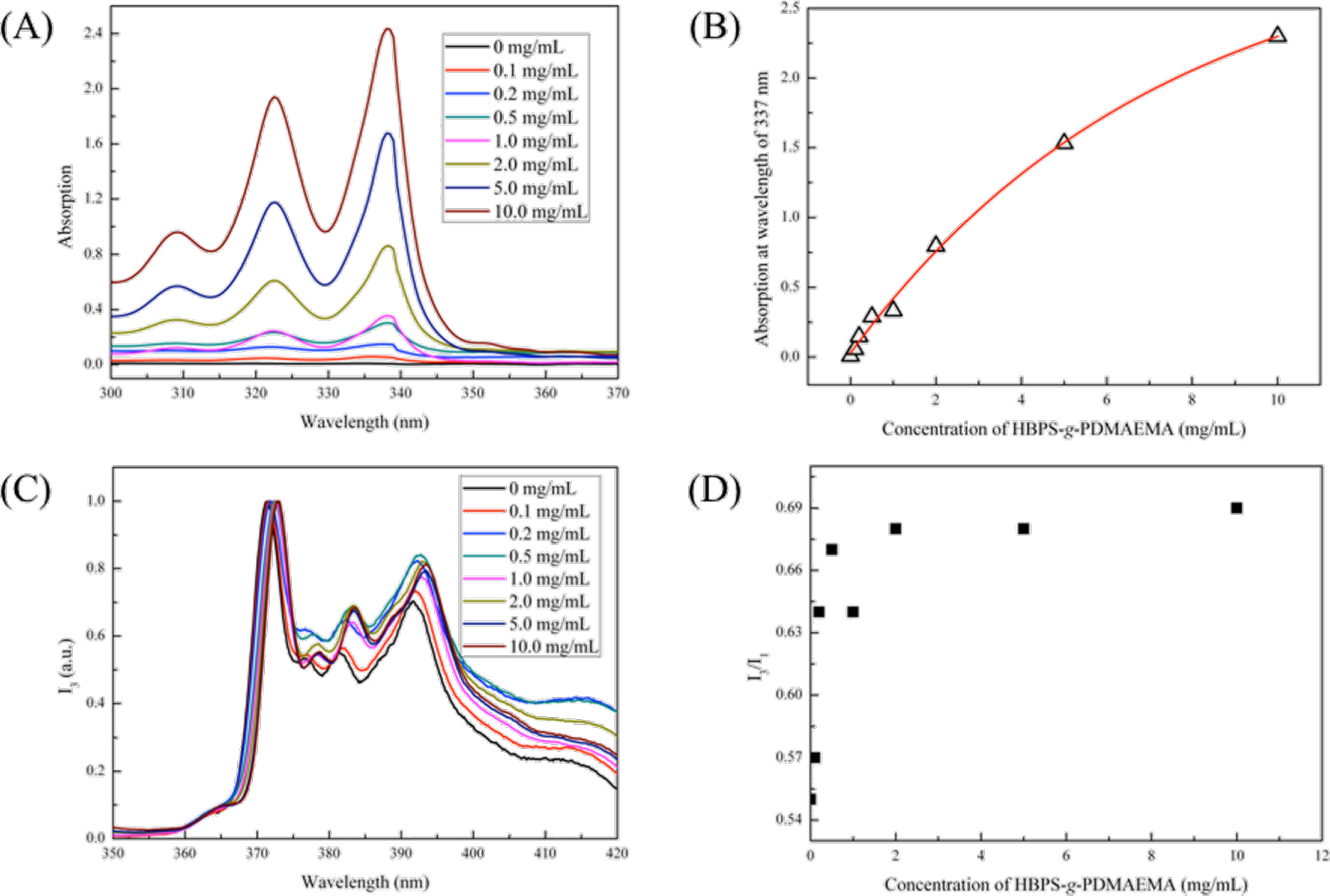
(A) The UV–vis spectra of HBPS-*g*-PDMAEMA/pyrene solution with different polymer concentrations; (B) the absorption at 337 nm of HBPS-*g*-PDMAEMA/pyrene solution with different polymer concentrations. (C)The fluorescence spectra normalized at 373 nm of HBPS-*g*-PDMAEMA/pyrene solution with different polymer concentrations and (D) the *I*_3_/*I*_1_ values of HBPS-*g*-PDMAEMA/pyrene solution with different polymer concentration.

To confirm the loading capability of HBPS-*g*-PDMAEMA aggregates for hydrophobic molecules, pyrene was used as a model molecule. Pyrene, a fluorescence-sensitive molecule, is often used for detecting the local polarity of amphiphiles. To prepare the pyrene/HBPS-*g*-PDMAEMA aqueous sample solutions, 5 to 6 mg pyrene was added in 5 mL HBPS-*g*-PDMAEMA solution with concentrations from 0 to 10.0 mg/mL followed by agitation for 2 h and filtration through a 0.45 μm micro-filtration membrane. Figure 7A,B shows UV–vis data below 25 °C. It was observed that the intensity of the peak attributed to pyrene around 337 nm was increased with the increase in the concentration of HBPS-*g*-PDMAEMA, which indicated that pyrene was transported to the aqueous solution by using HBPS-*g*-PDMAEMA.

The graft copolymer HBPE-*g*-PDMAEMA is an amphiphilic polymer containing a hydrophobic HBPS segment and hydrophilic PDMAEMA segments. The amphiphilic structure of this polymer affords the capability to disperse small hydrophobic molecules in aqueous solution. The ratio of *I*_3_/*I*_1_ from fluorescence spectroscopy, the intensity ratio between the peak around 382 nm and the peak around 373 nm, showed a relatively polar environment, with higher values meaning lower polarity. In this work, the pyrene/HBPS-*g*-PDMAEMA aqueous sample solutions were used for detecting the environmentally located polarity of encapsulated pyrene. Figure 7C,D showed that the intensity of *I*_3_ (normalized at *I*_1_) was increased to 0.68, as concentration of HBPE-*g*-PDMAEMA was increased to 2.0 mg/mL. Although this value is much lower than pure aliphatic hydrocarbons (1.65–1.8) [24], the improvement compared to water is a strong evidence for the loading capacity of HBPS-*g*-PDMAEMA for small hydrophobic molecules.

**Figure 8:**
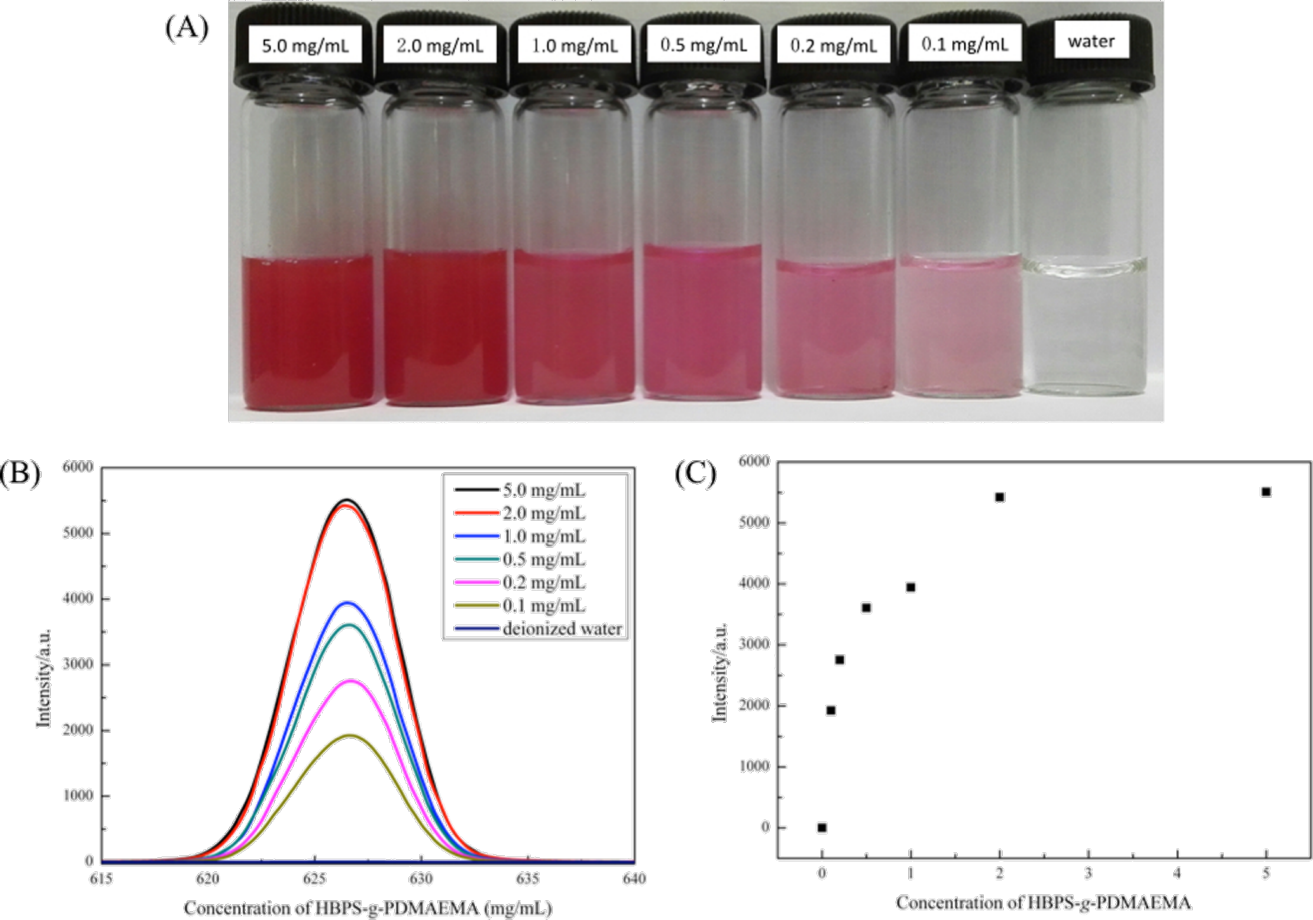
(A) Photographs of HBPS-*g*-PDMAEMA/NR in aqueous solution with different polymer concentrations; (B) the fluorescence spectra of aqueous HBPS-*g*-PDMAEMA/NR solutions with different polymer concentrations; (C) the fluorescence intensity of HBPS-*g*-PDMAEMA/NR in aqueous solutions with different polymer concentrations at peak around 627 nm.

To further confirm the capability of HBPE-*g*-PDMAEMA aggregates to encapsulate small hydrophobic molecules, Nile red (NR), a commonly used fluorescence dye was used as a model molecule. The preparation of HBPS-*g*-PDMAEMA/NR solutions was similar to HBPS-*g*-PDMAEMA/pyrene solutions. As shown in Figure 8A, the encapsulation of NR by HBPE-*g*-PDMAEMA aggregates can be observed by the naked eye. The solution containing these aggregates showed the pink color of NR in hydrophobic environment, and solutions without aggregates were colorless. These results indicated the successful encapsulation of NR in HBPE-*g*-PDMAEMA aggregates. The fluorescence spectra of HBPE-*g-*PDMAEMA/NR in aqueous solution shows peaks around 625 nm which were attributed to NR, as shown in Figure 8B. Figure 8C shows the fluorescence intensity of the peaks around 625 nm as a function of the concentration of HBPE-*g*-PDMAEMA aggregates, and it was found that a large amount of NR was dispersed in water with more HBPE-*g*-PDMAEMA aggregates. The trend was nonlinear unlike the linear trend of NR encapsulation in micelles, as the size and aggregation of HBPE-*g*-PDMAEMA are influenced by the polymer concentration as shown in Figure 3.

**Figure 9:**
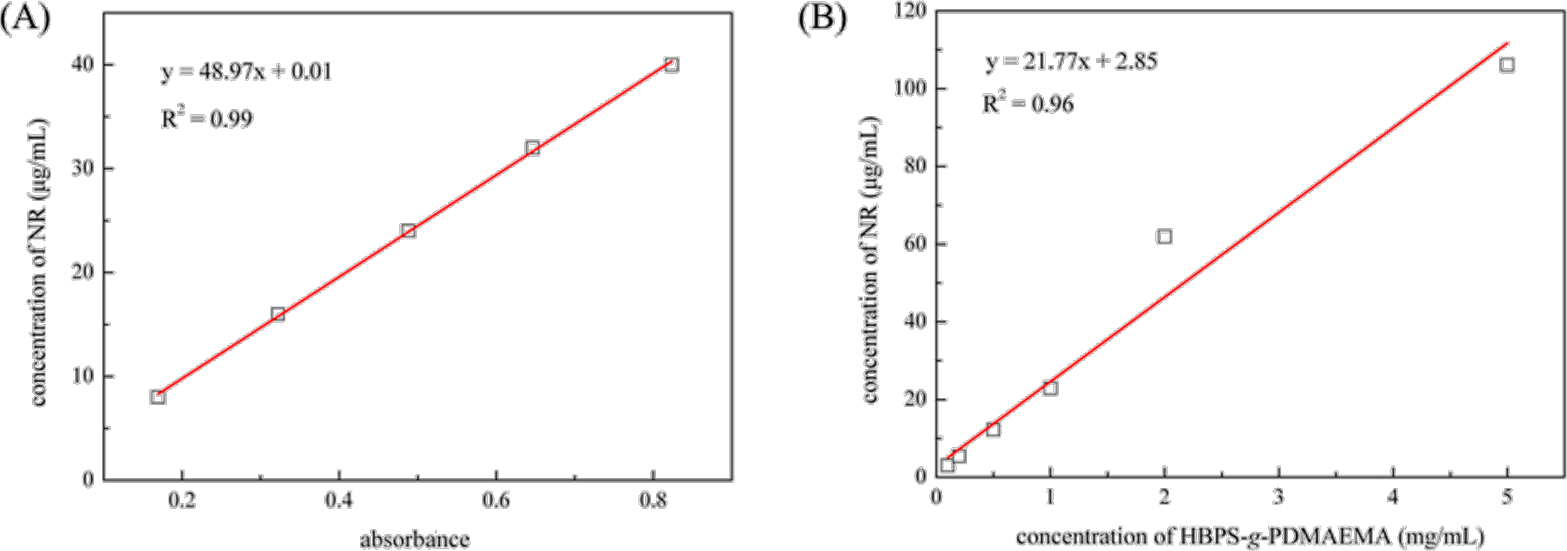
(A) Plot of the concentration of NR as a function of the UV–vis absorbance at a wavelength of 520 nm in dioxane. (B) Plot of the concentration of NR as a function the concentration of HBPS-*g*-PDMAEMA. (Red line is the best linear fit line used for studying loading capacity.)

To further investigate the loading capability for NR of HBPE-*g*-PDMAEMA aggregates in aqueous solution, a standard curve was drawn as shown in Figure 9A and the above mentioned HBPS-*g*-PDMAEMA/NR solutions were studied by UV–vis spectroscopy as shown in Figure 9B. The standard curve of absorbance at 520 nm of NR in dioxane exhibits an excellent linear relationship of which R^2^ is 0.99. By using the standard curve, the concentration of NR in each sample solution can be calculated. From calculation and fitting line, the result shows that 1 mg of HBPS-*g*-PDMAEMA could load about 28 μg NR, which is limited. It is maybe a result of low ratio of HBPS segments and this inefficient loading method. The R^2^ of the best linear fit line is 0.96 and it shows that the concentration of NR increasing linearly with concentration of HBPS-*g*-PDMAEMA. It demonstrates that HBPS-*g*-PDMAEMA is still intact up to a concentration of 0.1 mg/mL. Therefore, the lower critical aggregation concentration in water is beyond 0.1 mg/mL.

## Conclusion

In this paper, the graft copolymer HBPS-*g*-PDMAEMA was synthesized by ATRP and characterized by ^1^H NMR and GPC. The phase-inversion behavior of HBPS-*g*-PDMAEMA in aqueous solution was studied. Obvious optical changes were observed when the temperature reached the LCST. The influence of polymer concentration, ionic strength and pH value was also investigated. The size of the aggregates was affected by the corresponding stimulus. A higher temperature led to the shrinkage of PDMAEMA chains and resulted in the decrease in aggregates size. The polymer tends to further aggregation near the LCST at higher polymer concentrations. It was found that the basic environment results in narrow PDI of HBPS-*g*-PDMAEMA aggregates at a temperature above the LCST. In acidic environment, the HBPS-*g*-PDMAEMA aggregates showed no phase inversion. The HBPS-*g*-PDMAEMA aggregates/pyrene solutions were used to study the loading ability for small hydrophobic molecules. The results demonstrated that the HBPS-*g*-PDMAEMA aggregates were capable of encapsulating small hydrophobic molecules. The *I*_3_/*I*_1_ value reached 0.7 when the polymer concentration was above 2.0 mg/mL. The encapsulation of Nile red showed that HBPS-*g*-PDMAEMA aggregates are good containers for small hydrophobic molecules.

## Experimental

### Materials

All reactions were performed under argon and argon gas was purified by passing through four columns loaded with potassium hydroxide, 4 Å molecular sieves, silver molecular sieves and potassium/sodium alloy. Vinylbenzyl chloride (VBC) was purified by passing through a column packed with alkaline alumina. DMAEMA and benzyl chloride (BC) were used as received. Water was deionized before use. CuBr was purified by using acetic acid and methanol before use. Alkaline alumina, CuCl, 2,2-bipyrene (bpy) and *N*,*N*,*N*′,*N*″,*N*″-pentamethyldimethylenetriamine (PMDETA) were used as received.

### Preparation of HBPS and HBPS-*g*-PDMAEMA [26]

The synthetic routes to HBPS and HBPS-*g*-PDMAEMA are shown in Figure 1A. The synthesis of HBPS was done by using a Schlenk line system under argon atmosphere. The solution of monomer was prepared by the addition of 3.0 mL VBC (21.1 mmol) in 8.4 mL benzyl chloride followed by deoxygenation by three cycles of freezing–thawing–freezing. The Cu complex solution was prepared by dissolving 0.86 g CuCl and 2.72 g bpy in 8 mL BC in the absence of oxygen. Then 2.0 mL of Cu complex solution containing 0.21 g CuCl (2.1 mmol) and 0.68 g bpy (4.2 mmol) was added into monomer solution. The resulting reaction mixture was heated to 120 °C in oil bath with constant stirring. After 4 h, the polymerization was stopped by placing the resulting reaction mixture into liquid nitrogen and under ventilation. The solution was diluted with THF. To remove Cu complex, the diluted solution was passed through neutral alumina column. The eluted solution was concentrated and dropwise added in large amount of methanol. After filtration, white solid product was collected and redissolved in THF. The purified polymer was precipitated by adding resulting solution in large amount of methanol. This process was repeated and the obtained product was dried under vacuum at 40 °C.

HBPS-*g*-PDMAEMA was synthesized by using a Schlenk line system under argon atmosphere. The solution of Cu complex was prepared by dissolving 76.1 mg CuBr and 121.0 μL PMDETA in 7 mL anisole which was then deoxygenized by three freezing–thawing–freezing cycles. The initiator solution was prepared by dissolving 36.1 mg HBPS in 4.0 mL anisole. The monomer solution was prepared by dissolving 2.5 mL DMAEMA (14.9 mmol) and 1 mL of initiator solution containing 9.0 mg HBPS ([–Cl]_0_= 75 μmol which was calculated from ^1^H NMR) in 3.0 mL anisole. 1.0 mL Cu complex solution containing 10.9 mg CuCl (75 μmol) and 17.3 μL PMDETA (82 μmol) were added into monomer solution. The reaction mixture was heated to 90 °C in an oil bath under constant stirring. After 3 h, the polymerization was stopped by immersing the reaction mixture in liquid nitrogen and putting it under ventilation. The product was precipitated by dropwise addition of the reaction solution in a large amount of hexane. After filtration, the white product was collected and then redissolved in THF. In order to remove the Cu complex, the diluted solution was passed through a neutral alumina column. The eluted solution was concentrated and dropwise added in large amount of hexane to precipitate the product. This process was repeated and then the obtained product was dried under vacuum at 40 °C.

### Characterizations and measurements

The proton nuclear magnetic resonance (^1^H NMR) spectra were recorded on a Bruker 400M spectrometer. The molecular weight of polymer was determined by gel permeation chromatography (GPC) (Waters-Wyatt) equipped with RI, UV, viscosity and LS detectors using tetrahydrofuran (THF) as eluent. The UV–vis spectra were recorded on a UV3802 (UNICO) ultraviolet spectrophotometer. The size and zeta potential of the polymer in aqueous solution at different temperature were measured on a Zetasizer 3000HSA dynamic light scattering (DLS)/zeta potential analyzer. The fluorescence spectra were recorded on an F-280 (Tianjin Gangdong) fluorescence spectrophotometer.
